# Is the policy of allowing a female labor companion feasible in developing countries? Results from a cross sectional study among Sri Lankan practitioners

**DOI:** 10.1186/s12884-017-1578-z

**Published:** 2017-11-22

**Authors:** Hemantha Senanayake, Rajitha Dilhan Wijesinghe, Kesavan Rajasekharan Nayar

**Affiliations:** 1 Department of Obstetrice & Gynaecology, Faculty of Medicine, University of Colombo, Colombo, Sri Lanka; 2 Professorial Obstetric Unit, De Soysa Maternity Hospital, Colombo, Sri Lanka; 3 Global Institute of Public Health, Trivandrum, Kerala India

**Keywords:** Labor companion, Doula, Labor support, Labor

## Abstract

**Background:**

Companionship during labor is known to have both physical and psychosocial benefits to mother and baby. Sri Lanka made a policy decision to allow a labour companion in 2011. However, implementation has been unsatisfactory. Given the leading role Obstetricians play in the implementation of policy, a study was undertaken to assess the knowledge, attitudes and practices among them.

**Method:**

A descriptive cross sectional study was conducted among consultant obstetricians working in the state hospitals using the platform ‘Survey Monkey’.

**Results:**

Out of the 140 consultant obstetricians invited, 68(48.5%) participated. Among the study participants, 40 (58.8%) did not allow labour companions in their wards. Lack of space (*n* = 32; 80%) and the volume of work in the labor wards (*n* = 22; 55%) were the commonest reasons for not allowing a companion. Only 16.7% (*n* = 5) of the obstetricians handling more than 300 deliveries per month allowed a companion (*p* = 0.001). Less than 50% of the obstetricians were aware of the advantages associated with the practice such as shorter labor, lesser analgesic requirement, higher chances of a normal birth, improved neonatal outcome and reduced requirements for labor augmentation for slow progress of labor. Knowledge on advantages on breast feeding and reduced need of instrumental delivery also remained low.

**Conclusion:**

In an individual unit, the consultant often decides policy. The study points out the need to improve awareness among the practitioners.

## Background

Giving birth is one of the most important events of a woman’s life. It is a sentinel event for her family. The process of giving birth must not be considered a mere biological event, but a process that is associated with many social and emotional connotations. It is a right of every woman to be supported and to receive the most up to date, evidence based care during childbirth.

Modern times have seen increasing medicalization of the management of labor [1]. It seems that emphasis is now on safety at the expense of its emotional aspects. Historically, laboring women have received support from other women of their social or family circle, a practice that has been used across cultures almost universally. These aspects have become marginalized as a result of concentrating services on safety.

Sri Lanka is no exception to this phenomenon. Successive governments over the past six decades have followed a policy of increasing access to and improving the quality of healthcare. The policies have had a heavy focus on infrastructure development and investment in skilled attendance at birth. These initiatives included encouraging women to deliver in hospitals, rather than at home. Over 99% of women now receive skilled attendance at birth [2–4]. Maternal death rates have been brought down from 61/100,000 live births in 1995 to 32.03/100,000 in 2014 [4].The public sector, which provides free healthcare to all Sri Lankans handles 94.6% of the births that occur in the country [4].Since its achievements in health indices are in keeping with countries that have ten times its gross domestic product, Sri Lanka is held out as model for non-industrialized nations [5].

As a corollary to these, women have had to pay the price for medicalization of birth. The vast majority of women who deliver in public sector hospitals in Sri Lanka will deliver in labor wards that are out of bounds to ‘outsiders’. In effect this means that some women will be deprived of contact with their immediate family and social circle on one of the most important days of their lives. Authors have expressed concern regarding dehumanization of the process of birth as a global issue [6].

A person who provides non-medical physical, emotional and informational support during and after labor is known as a ‘Doula’. Almost every culture has had its own version of Doulas, which had been practiced for thousands of years. Provision of continuous care during labor can be conducted either by hospital staff, a woman from outside the laboring mother’s social and family circle or by a companion of the woman’s choice from her family or social circles. In the modern obstetric practice, male partner’s involvement in labor support is frequently seen. However, some evidence shows that male companions may not confer major advantages to outcomes [7].

Non-medical support during labor has shown positive intrapartum, perinatal and neonatal outcomes [8]. A Cochrane review analyzing 22 trials involving 15,288 women from 16 countries demonstrated clear benefits on increased vaginal birth rates, fewer requirements of analgesics, shorter labors and improved maternal satisfaction. The review concluded that in view of clinical benefits to both mother and infant, all women should have continuous support throughout labor [6]. In a review, the American Journal of Obstetrics & Gynecology has classified companionship during labor as one of the most effective interventions [9].

A randomized controlled trial conducted in Sri Lanka to assess the effectiveness of a female labor companion in the local setting corroborated these positive outcomes in the local setting. Continuous support by a female companion was associated with significant maternal satisfaction with labor and better establishment of breastfeeding within first 24 h. It also showed a statistically not significant reduction in the requirement for augmentation of labor [10].

Despite such compelling evidence, women in less privileged settings are deprived of this valuable intervention. Thus, at a time of great psychological demand, women have no choice but to endure labor without contact with anyone from their immediate social circles. The World Health Organization recommends labor companionship as a low cost intervention to improve outcomes of labor [11]. In keeping with this, Sri Lankan government has made a policy to allow a labor companion [12]. However, implementation has been unsatisfactory. Ironically, in Sri Lanka, the norm in private hospitals is to allow a companion of the mother’s choice, including the male partner.

In Sri Lankan government hospitals, the labor rooms are designed to accommodate multiple labor beds that are separated by retractable curtains. Most labor wards will therefore not provide a high level of privacy, precluding the facility of allowing a male labor companion. However, at the presently available level of infrastructure, Sri Lankan labor rooms will be able to accommodate female labor companions without the requirement of significant changes.

In 2014, nearly 95% the deliveries were conducted in government hospitals that had the services of a Consultant Obstetrician [4]. Consultant Obstetricians & Gynaecologists are Board-Certified specialists who have completed 6 years of structured postgraduate training in the specialty. Board Certification is a prerequisite to holding a Consultant post in public sector hospitals in Sri Lanka. Polices on patient care at the local level are influenced heavily by the Consultant in charge. This is true for the practice of allowing a female labor companion as well. One of the main limiting factors against wider acceptance of allowing a female labor companion may be their lack of acceptance of its advantages. An initial resistance is natural for any change in the existing practice.

Given the leading role of Obstetricians play in the implementation of policy, we undertook a study to assess the knowledge, attitudes and practices with regard to female labor companionship. We invited all the Consultant Obstetricians providing care in government hospitals to participate. This would be useful in understanding the constraining and facilitating factors and would help in expanding this service.

## Method

The Ethics Review Committee of the Faculty of Medicine, University of Colombo, granted ethical clearance for the study. Only consultants currently providing obstetric care in state hospitals were included in the study. A list of all the consultant obstetricians employed in the state hospitals was prepared using a list of email addresses obtained from the Sri Lanka College of Obstetricians and Gynecologists (SLCOG).Without exception, all specialists who provide obstetric care in state hospitals are members of the SLCOG. Thereby we were able to cover all the obstetricians &hospitals in the state sector.

A self administered questionnaire was developed aiming to assess participant’s characteristics, physical facilities available in their hospitals, workload, obstetricians’ knowledge on benefits of companionship, their opinions on the possible benefits and drawbacks of labor companionship, reasons for not allowing a companion in their labor rooms and experiences of those who already practice this intervention. Questions on the last three components included both closed and open-ended questions. In assessing the knowledge on the benefits of companionship, a score of more than 4 out of 7 was considered as satisfactory. The questionnaire was pre tested among postgraduate trainees in obstetrics and gynaecology.

We distributed the questionnaire via e-mail, as the targeted study participants were spread throughout the country. We included Information regarding the survey in the email and conducted the survey using the platform ‘SurveyMonkey’. This platform allowed us to collect individual responses from each participant. Data was collected over a period of 1 month. Consultants who returned the questionnaire were considered to have given implied consent for the research. We maintained anonymity regarding the identity of consultants and the data generated.

Data was analyzed using a computer based statistics package. Pearson Chi square test was used for the calculation of probability values in the majority. “Likelihood ratio chi square test” was used when there were cells with count less than 5. Alpha was set at 0.05.

## Results

Out of the 140 consultant obstetricians invited, 68(48.5%) participated in the survey. The survey completion rate was 44.3%(*n* = 62). Table 1 demonstrates the profile of study participants and their labor wards.

**Table 1:** Profile of study participants and labor wards

Age of Obstetrician	Number of practitioners
Below 40	9 (13.2%)
41 to 50	30 (44.1%)
51 to 60	21 (30.9%)
More than 60	7 (10.3%)
Missing	1 (1.5%)
Total	68 (100%)
Experience of the obstetrician	
Less than 5 years	2 (2.9%)
6 to 15 years	30 (44.1%)
16 to 25 years	19 (27.9%)
More than 25 years	15 (22.1%)
Missing	2 (2.9%)
Total	68 (100%)
Number of deliveries (per month)	
Less than 100	5 (7.4%)
100 to 200	11 (16.2%)
200 to 300	17 (25%)
More than 300	30 (44.1%)
Not responded	5 (7.4%)
Total	68 (100%)
Number of Labor room beds	
1 to 3	5 (7.4%)
4 to 6	17 (25%)
7 to 10	21 (30.1%)
More than 10	20 (29.4%)
Missing	5 (7.4%)
Total	68 (100%)
Separation of beds	
Yes	48 (70.6%)
No	15 (22.1%)
Missing	5 (7.4%)
Total	68 (100%)
Shared labor room with other Obstetricians	
Yes	40 (58.8%)
No	23 (33.8%)
Missing	5 (7.4%)
Total	68 (100%)

Table 2 shows participants’ knowledge regarding the evidence-based medical benefits of allowing a female labor companion.

**Table 2:** Participants knowledge on the benefits of a female labor companion

Benefits	Number of practitioners
Improved moral support to mothers	61 (96.8%)
Improved care by health workers	48 (76.2%)
Advantages to establishment of breast feeding	39 (61.9%)
Reduced instrumental and caesareans deliveries	33 (52.4%)
Shortened length of labor	32 (50.8%)
Reduction in the need of augmentation	28 (44.4%)
Improve neonatal outcome	27 (42.9%)

Multiple responses were accepted for this question. 63 participants responded for the question

Even though the majority of the obstetricians were knowledgeable on the benefits on improved care provision and moral support, positive effects on the progress of labour and neonatal outcome were not known to a majority.

Table 3.demonstrates possible disadvantages of a labor companion as cited by the participants.

**Table 3:** Possible disadvantages of allowing a female labor companion

Possible disadvantages	Number of practitioners
Breach of privacy of other mothers	46 (71.9%)
Possible chance of complaints/litigation	44 (68.7%)
Possible interference to routine medical care	41 (64.0%)
Risk of theft	23 (35.9%)

Multiple responses were accepted for this question. 64 participants responded to the question

Among the study participants, 40 (58.8%) did not allow labor companions in their wards. We explored the reasons for not allowing a companion. Results are shown in Table 4.

**Table 4:** Reasons for not allowing a female labor companion (among obstetricians who do not allow a female labor companion)

Reason	Number
There isn’t adequate space between beds	32 (80%)
The labor ward is too busy	22(55%)
Nursing/ midwifery staff not agreeable	17 (42.5%)
There are no curtains separating the beds in the labor room	12 (30%)
Population that I am serving is not capable of using this kind of service	12 (30%)
Hospital administration is not agreeable	10 (25%)
I do not believe this is advantageous in Sri Lanka	5 (12.5%)

Multiple responses were accepted for this question. 40 participants responded to the question

The problem of inadequate space is the most frequently pointed out reason by the respondents followed by the heavy workload they have to handle. Analysis of open-ended questions reveals that there is lack of awareness among the practitioners regarding the benefits of allowing labour companions. Resource constraints and non-availability of technologically advanced facilities in hospitals was also a serious problem as this precludes provision of ideal care under normal and emergency situations. Respondents felt that technological advancement and provision of quality services such as adequate resources and personnel are the priorities compared to allowing labor companions. Overcrowding in labor wards due to the presence of outsiders was a problem pointed out by many respondents. Respondents also perceived possible interference in patient care by the companion during the process of delivery as a negative factors in allowing labor companions.

However, respondents also perceived the positive aspects of allowing a labor companion. Respondents felt that women who received this support were happy and contented. It also led to higher rates of vaginal delivery and reduction in caesarian sections. Some respondents also considered the companions as the first evidence against complaints from clients. Individual responses to the open questions on advantages and disadvantages experienced by the consultants who are already allowing companionship are shown in Table 5.

**Table 5:** Advantages/Disadvantages experienced by the units that allow a companion- Responses to open questioning

Advantages	Disadvantages
1. ‘Woman is very comfortable with a known person by her side’ 2. ‘In addition to above mentioned, can easily convince a primi asking for cesarean section to deliver vaginally’ 3. ‘Women who receive this intervention are happy’ 4. ‘Less shouting, shorter labor, more normal delivery, satisfied mothers, satisfied staff’ 5. ‘increased maternal satisfaction’ 6. ‘The woman would be relaxed and confident’ 7. ‘highly satisfied consumers, They are the 1st evidence against complaints.’ 8. ‘extreme satisfaction to mother and her husband/staff not aggressive -overall it is very good for staff health and duty of care’ 9. ‘reduce anxiety of patient leading to better progression of labour’	1. ‘Govt. hospitals lack proper facilities’ 2. ‘Overcrowding of antenatal & postnatal wards as companions stay in hospitals well before established labour& especially after delivery and become nuisance to the staff’ 3. ‘resistance from hospital administrators, specialists, other junior doctors, some members of the nursing and midwifery staff’ 4. ‘Potential interventions to patient management’ 5. ‘inadequate staff / expensive / much objections from staff initially, but got used to it’

Table 6 demonstrates the associations of allowing a female labor companion with clinician and labor ward characteristics.

**Table 6:** Associations of allowing a female labor companion with clinician and labor ward characteristics

Variable characteristic	Labor companion		Significance
	Yes	No	
Age of Obstetrician			
Less than 40 years	1(14.3%)	6(85.7%)	*P* = 0.429
40 to 50 years	12(40%)	18(60%)	
More than 50 years	10(38.5%)	16(61.5%)	
Experience as Consultant Obstetrician			
Less than 15 years	11(34.4%)	21(65.6%)	*P* = 0.853
16 to 25 years	6(35.3%)	11(64.7%)	
More than 25 years	6(42.9%)	8(57.1%)	
Obstetricians knowledge on evidence-based advantages of labor companionship^a^			
Satisfactory	18(54.5%)	15(45.5%)	P = 0.001
Unsatisfactory	4(13.8%)	25(86.2%)	
Number of deliveries			
Less than 200	12(75%)	4(25%)	*P* < 0.001
200 to 300	6(35.3%)	11(64.7%)	
More than 300	5(16.7%)	25(83.3%)	
Number of Labor room beds			
1 to 6	11(50%)	11(50%)	*P* = 0.264
7 to 10	6(28.6%)	15 (71.4%)	
More than 10	6(30%)	14(70%)	
Separation of beds			
Yes	23(47.9%)	25 (52.1%)	P < 0.001
No	0(0%)	15(100%)	
Shared labor room with other Obstetricians			
Yes	11(27.5%)	29(72.5%)	*P* = 0.05
No	12(52.2%)	11(47.8%)	

^a^ Based on the questionnaire

## Discussion

Following the successful trial in 2008, Sri Lanka government included allowing a labor companion to the ‘National Strategic Plan for Maternal and Newborn Health’ in 2011 [12]. Even though state health authorities made this policy decision, it has been a failure, since most obstetricians do not follow it. While data on birth companionship in Sri Lanka is lacking, among those who completed the questionnaire, 40 (58.8%) did not allow it in their units. It is also worth noting that 51.4%(*n* = 72) did not respond to the invitation. Lack of interest in the intervention may be a reason for the high non-response rate. It could be reasonable to assume that the majority of non-respondents were not supportive of allowing a labor companion. Considering the fact that 55.7% did not complete the questionnaire, the proportion that allowed a companion is probably even less than the above figure.

In an individual unit, a policy decision on allowing a labor companion is dependent mostly on the consultant in charge. Many factors seemed to affect these decisions. Some of these were work related while others seemed more personal.

Inadequate space and heavy work load were the commonest reasons for not allowing a labor companion from the obstetrician’s point of view. With less than 100 hospitals in the country providing specialist obstetric care for approximately 320,000 deliveries per year [4], most obstetric units in the government sector are extremely busy. More than 50% the respondents were handling in excess of 300 deliveries per month. Only 16.7% (*n* = 5) of the obstetricians handling more than 300 deliveries per month were allowing a female labor companion. This relationship was statistically significant (*p* = 0.001). Similarly, units without partitioning between labor beds were not allowing a labor companion. This might be due to obvious issues regarding privacy. However, partitioning of labor beds can be accomplished at a low cost. The main rationale of allowing female labor companions rather than husbands was the lack of privacy in the labor rooms.

Personal factors were mainly related to the consultant’s knowledge and attitudes regarding labor companionship. According to Table 6, those who allowed female labor companions in their units had better knowledge on its evidence-based medical benefits. However, the temporal relationship between the knowledge and allowing a companion is difficult to assess. Interestingly less than 50% of the obstetricians were aware of shorter labor, the improved neonatal outcome and reduced rates of augmentation for slow progress of labor that results from allowing a labor companion. Knowledge on advantages on breastfeeding and reduced need of instrumental delivery also remained low. This reflects an overall knowledge gap on evidence-based advantages of labor companionship. Possibly, some obstetricians were not allowing female companions simply due to unawareness of the practice.

Interestingly, more than 25% of the obstetricians believed that the population they were serving is not capable of using such a facility. Around 10% did not believe that labor companionship is advantageous for the local population. In a similar manner, health workers in Zambia believed that birth companions would interfere with their work by giving traditional medicine [13]. However, experience in units that allowed a companion has been otherwise. Most of the women and the relatives were grateful to the medical staff for the opportunity of supporting and accompanying their loved ones during labor.

Among the obstetricians allowing birth companions, none complained of interference in medical management by the companions. Positive outcomes overwhelmed the negatives. Many obstetricians highlighted the increased positive attitudes towards vaginal delivery and increased satisfaction regarding medical care. In contrast to popular beliefs, most of the negative comments seemed trivial, such as increased crowding and resistance from some staff members. Thus, most reasons given by the obstetricians against labor companionship did not seem to be substantiated by real world scenarios.

Due to the scattered nature of the study population, email was used to distribute the questionnaires. This may be one of the drawbacks of the study. Background conditions such as allocated time to fill the questionnaire and external influences could not be controlled. There was room for the participants to refer to literature before answering the questionnaire, errors that are inevitable in an internet based study.

## Conclusion

In conclusion, the study points out the need to improve awareness among the practitioners, probably by workshops and continuous feedback mechanisms. We would suggest following interventions to promote the practice of allowing a female labor companion in Sri Lanka.

- Improvement of the Obstetrician’s knowledge on the benefits of allowing a female labor companion
- Creating a stage for obstetricians to share their experiences with each other
- Empowering women to request a labor companion
- Educating the public using mass/social media and to ensure privacy of all laboring mothers.
